# The Role of Social Media in Building Pandemic Resilience in an Urban Community: A Qualitative Case Study

**DOI:** 10.3390/ijerph20176707

**Published:** 2023-09-04

**Authors:** Joel Oommen George, Suzanne Elayan, Martin Sykora, Marin Solter, Rob Feick, Christopher Hewitt, Yiqiao Liu, Ketan Shankardass

**Affiliations:** 1 Centre for Information Management, Loughborough Business School, Loughborough University, Loughborough LE11 3TU, UKm.d.sykora@lboro.ac.uk (M.S.);; 2 School of Planning, University of Waterloo, Waterloo, ON N2L 3G1, Canada; 3 Department of Health Sciences, Wilfrid Laurier University, Waterloo, ON M5B 1W8, Canadakshankardass@wlu.ca (K.S.)

**Keywords:** social media, digital epidemiology, urban health, mental health, resilience

## Abstract

This paper explores the influence of social media in fostering resilience within an urban spatial context, specifically in Bangalore, India, during the COVID-19 lockdown, a period marked by a surge in digital communication due to movement restrictions. To control the rapid spread of the virus, over 1.38 billion people were given stay-at-home orders by the government of India during the onset of the pandemic. The restrictions in movement forced individuals to shift to online modes of connection and communication. As the field of digital epidemiology, that is, the use of digital tools and data to understand and improve health took center stage during the pandemic, the focus shifted towards the social media landscape, which is often associated with its negative aspects, such as misinformation. However, this paper delves into social media’s potential to build resilience on a local scale, particularly given its increased usage during the pandemic. Through in-depth online interviews with eight urban residents, we conducted a thematic analysis to understand social media’s role during the lockdown. Results indicate that social media facilitated effective information exchange and fostered a sense of community. Furthermore, it engendered an environment conducive to prosocial behavior, a known resilience amplifier. We also highlight the importance of baseline context regarding the users directly engaged in social media data generation with respect to digital epidemiology analytics tools for large-scale social media data and the need for qualitative input feeding into their design. Our study highlights the need for a balanced perspective on social media use in times of crisis, recognizing its potential to boost community resilience in an urban setting, and further enriching digital epidemiology approaches.

## 1. Introduction

‘Lockdown’ was declared the word of the year in 2020 by Collins Dictionary [1] as it poignantly summarized the collective experiences of billions. While crucial and necessary in stopping and slowing the spread of COVID-19 [2,3], numerous studies have since found that this collective experience has created a negative impact on mental health across all strata of society [4,5,6], while disproportionately affecting urban populations [7,8,9]. There were reports of directly linked suicides [10], stress, paranoia, and high levels of anxiety [11,12,13,14], including problems with sleeping [15] and access to healthcare [16], while more broadly the pandemic has resulted in a momentous loss of both economic and social resources [17,18].

The restricted movement caused by social distancing and isolation orders had resulted in a sharp spike in social media usage [19,20,21]. In 2020, a study by the GlobalWebIndex reported a rise of 10.5% in social media use when compared to the previous year, and over 40% of adults reported spending more time on social media [20]. During the same period, messaging rates increased by 70% globally across popular platforms such as WhatsApp, Facebook, and Instagram [21], while TikTok grew by several hundred million users over the pandemic to 1 billion active global users by September 2021 [22]. In a public letter, the dean of the Boston University School of Public Health, Sandro Galea [23], pointed out, “*COVID-19 is the first global pandemic of the social media age, the first of the ‘alternative facts’ era, and is occurring at a moment when politics and society seem to be in a state of accelerated flux*”, and the WHO had even dubbed it an “infodemic” [24], with streams of mis/disinformation invading our homes through online platforms [25]. The negative consequences of social media on this Information ecology were repeatedly and consistently highlighted across the literature [25,26,27,28]. While these concerns are of utmost importance, the positive influences and potential for social media to contribute to resilience, that is, the ability to adapt positively, drawing on internal and external resources, after experiencing adversity to regain or maintain mental well-being [29], cannot be ignored nor under-estimated [17,30,31]. There have been numerous studies that also leveraged social media content and footprints of related big data and their large-scale analysis to uncover topical themes and online as well as offline places of interest or concern [32] following major crises across urban areas [33,34], including during the COVID-19 pandemic specifically [35], such as the social media geographic urban stress and emotion detection tool presented in [36].

Such studies have allowed the collation and analysis of important user-generated datasets for substantive insights and findings of direct relevance to post-disaster mental health and public health in general [32,33,34,35,36,37], including, in this special issue, Gutierrez and colleagues [38], whose paper was dedicated to reproducibility and integrity of big data research in this context. Yet, there has been an overall lack of non-explorative [37] and in-depth qualitative studies involving the users directly engaged in social media data generation, and their motives and resilience-building behaviors, within this context. Without a better understanding of these processes and efforts to combine ‘small’ and big data [39], upstream research tasks have arguably limited interpretative scope, and digital epidemiology tools such as those found in [36] may lack important baseline context.

Hence, this study aims to uncover the social media-specific resilience-building factors within an urban community. We focus our investigation on community members from Bangalore, who were substantively affected by the pandemic lockdown. Our study seeks to understand the role of social media in related information sharing, prosocial, and primarily its resilience-building behaviors (see Section 1.1). Specifically, we focus on three related research questions:

*RQ1: Was social media effective in sharing health information during the lockdown period of the pandemic?*

The objective of the study is to analyze the type of information shared during the lockdown and to investigate the effectiveness of the shared information. The study takes into consideration the presence of misinformation online and seeks to analyze its effect on members of the community.

*RQ2: Did social media improve connectedness during the lockdown period of the pandemic?*

The objective of the study is to understand how members of the community connected with friends and loved ones during the lockdown. It seeks to investigate the role of social media in improving connectedness.

*RQ3: Did social media build resilience during the lockdown period of the pandemic?*

The objective of the study is to understand if social media was useful in building resilience during the lockdown. The study aims to investigate the factors that lead to building resilience and, therefore, extend the literature on pandemic recovery and large-scale future studies, where these could allow the integration of social media in future emergency response and recovery.

More broadly, our study contributes towards filling the paucity of research on social media being used as a tool for building resilience, and the threads that relate to this with respect to the already introduced context. As prior studies focus on the negative impacts of social media and/or those who are excluded from it, by choice or circumstances, this paper purposely examines participants who could be most affected (tech-literate daily users) and considers positive impacts to gain a better understanding about how social media is used during a time of crisis, and in the context of an understudied geographic region.

Next, we introduce further key background literature on resilience and social media use, with a focus on the Indian context directly related to our empirical study.

### 1.1. Background on Resilience and Social Media Use during the Pandemic

The term resilience has many contexts in which it has been defined. For one, as already stated, resilience is the ability to adapt positively after experiencing adversity to regain or maintain mental well-being [29]. At a broader level, Meerow et al. [40] refer to urban resilience as the ability of an urban system, and all its constituent socio-ecological and socio-technical relationships to maintain or rapidly return to desired functions in the face of a disturbance, to adapt to change and to quickly transform systems that limit current or future adaptive capacity. Resilience has also been used to describe the ability of persons, communities, and the public at large to adapt and persist despite change [41], while disaster resilience is the capacity of people, communities, and society to absorb, resist, and recover from the effects of a hazard with efficiency and timeliness [42].

Highly relevant to our study, Walsh [43] introduces a framework for COVID-19-specific family resilience, in which they identify three important processes of resilience: (a) meaning making, (b) positive outlook, and (c) finding purpose. The first process of meaning making is explained by Coutu [44] and it involves the ability to find meaning in one’s environment during a difficult time. The second process is supported by Kaye-Kauderer et al. [45], who state that staying positive despite negative circumstances can help alleviate some of the negative emotions caused by the pandemic. The creative use of social media can help with this process, especially during lockdown periods [46]. The third process is supported by López et al. [47], who observed that there is a strong association between people who had a sense of purpose and increased resilience. Therefore, it is of substantive relevance to understand whether some people are more resilient than others during a situation of crisis. Bono, Reil, and Hescox [48], for instance, argue that a higher resilience is seen among those with more ‘grit’. 

A meta-analysis by Hu, Zhang, and Wang [49] shows that increased resilience is directly related to positive indicators of mental health such as satisfaction with life. Past studies have also looked at the role of personality in influencing overall well-being [50,51] and the link between the big five personality (openness to experience, conscientiousness, extraversion, agreeableness, neuroticism) traits to perceived resilience. The results indicate a negative association between resilience and neuroticism, and conversely a low to moderate positive association between resilience and the other four personality traits [51]. While these studies search for resilience within personality traits, other studies suggest resilience can be built through engaging in resilience and empowering processes [31,52], including activities such as physical exercise and following a healthy diet, as well as empowering social media use [53,54]. 

While the studies above address factors influencing resilience from an individual perspective, other studies have explored the factors that influence the resilience of the community at large. Magis [55] argues that resilience in communities can be evidenced by their ability to adapt to change and thrive in conditions of crisis. Cutter et al. [56] add that community resilience is not just reliant on a single system of operation but rather on a co-existence of multiple integrated systems. The importance of having integrated systems is ever prominent in the case of urban environments, and such settings offer various opportunities for several social and economic amenities [55,57,58]. Aldrich and Meyer [59] also stress the importance of social capital in disaster recovery. Aldrich [60] argues that social capital is one of the key influences on the pace of recovery in the aftermath of a disaster. Studies in the past have shown that social media can improve social capital [61] and newer studies suggest that social media plays a crucial role in increasing social capital during isolation [62]. In addition to fostering social capital and playing a modulating role in resilience, social media can also encourage prosocial behavior during times of crisis. Prosocial behavior is defined as the voluntary actions of kindness, compassion, and helping behavior that can benefit another [63]. 

Taking a closer look at the case of urban settings across India, various public events such as election rallies and religious gatherings resulted in a sharp spike in COVID-19 cases across the country [64]. With over 300,000 cases reported per day, a shortage in intensive care beds and medical oxygen plagued the hospital systems unequipped to handle the high number of cases. Oxygen demand was at 700% in certain parts of the country and help was offered from around the world including the United States, United Kingdom, and UAE [65]. 

A silver lining during the collapse of the hospital systems was the abundance of help in the form of crowdsourcing and crowdfunding via social media channels such as Twitter, Facebook, WhatsApp, and Instagram [66]. A study by Faruqui et al. [67] reports the use of social media by overwhelmed hospitals to locate suppliers of oxygen. Social media was also used to raise funds for families that could not afford treatment during this time. Instagram hashtags such as ‘#hospitalbedsinpunjab’ and ‘#hospitalbedsindelhi’ were used to track the status of beds in both public and private hospitals via Instagram, while the hashtag #oxygenbedsleads was used to keep track of oxygen manufacturers, turning social media into an unofficial disaster management tool; citizens used these platforms to help source crucial resources such as hospital beds, cylinders, and plasma [68]. 

Studies in the past have postulated that prosocial behavior stems from people who view a shared identity with someone going through a hard time [69] and have shown that people who engage in prosocial behaviors have an improved sense of well-being and life satisfaction [70,71]. Aresi et al. [69] argue that prosocial acts improve the resilience of a community as they promote a sense of meaning and purpose. Thus, forthcoming public health threats can be better moderated with the integration of social media as a tool for response, recovery, and preparedness [72,73].

As evidenced, past studies have shown the importance of social media in disaster response and the low cost, wide reach, and timely dissemination of both non-official and official sources of information via social media following a disaster, as well as helping to improve connectedness and help build and co-create resilience. 

## 2. Materials and Methods

### 2.1. Study Site

The urban community selected in this study was the city of Bangalore, the capital of southern Karnataka state, and with just over 12 million inhabitants, is the third most populous city in India. Bangalore is also known as the ‘Silicon Valley of India’ with high levels of digital literacy [74,75]. At the apex of the pandemic, the Bangalore Urban district held the unfortunate distinction of being India’s hardest-hit area [76], recording the nation’s highest count of active cases, surpassing 150,000 cases [77]. Considering the aforementioned factors, Bangalore was deemed a suitable urban context for examining the role of social media in fostering pandemic resilience.

### 2.2. Sample

We employed a purposive sampling approach in selecting interviewees, ensuring that they were suitable candidates with respect to the objectives of our study [78], characterized by having been residing in the Bangalore urban district during the lockdown, being active members of their neighborhoods, having some digital literacy, and having used social media actively during the lockdown. All eight participants were employed and university educated at the time of the interviews, with two participants working in IT, two participants in banking and finance, one participant as a lawyer, one participant as a senior manager in one of the big four consulting firms, one participant as a strategy and insights manager, and one participant who was self-employed. These individuals, by virtue of their professions and educational backgrounds, had a higher degree of engagement with digital tools, including social media—a key element of our study. Secondly, given the qualitative nature of our study, following the interpretivist epistemological research philosophy rooted in the qualitative research tradition [79,80], our focus was on obtaining in-depth, rich, and contextually informed views (DiCicco-Bloom and Crabtree, 2006) 80 rather than aiming for generalizability. Carminati [81] (p. 2096) further describes the definition of generalizability, and how interpretivism emphasizes comprehending human actions rather than forecasting or generalizing the underlying causes and outcomes. Hence, the small sample size of eight participants is justified as it allows for a detailed exploration of each individual’s experience and perceptions, thereby providing valuable insights into our research questions. We were originally aiming for six to ten participants; however, the final number of interviewees was directly related to reaching saturation, that is, when no new perceived, indicative themes or insights were emerging from the data, suggesting that additional interviews were unlikely to provide significant new information [79]. This helps ensure that the research captures a comprehensive understanding of the phenomenon under study. Individual, semi-structured, in-depth interviews [81,82,83] were conducted, with a focus on capturing rich in-depth information about the participant’s perspective and experience. In line with DiCicco-Bloom and Crabtree [81], our primary focus was on the person interviewed becoming more of a participant in meaning making than simply a conduit from which information is retrieved, thus allowing the exploration of deeper and more complex meaning making. We conducted all interviews online via standard video-call software [82,84,85] over a period of ten days and experienced no issues with the quality of these video-calls nor with the internet connection/bandwidth. The interviews followed a semi-structured design and made use of open-ended questions that had predetermined talking points [81] and allowed for a more consistent while still flexible and versatile approach to eliciting responses from interviewees [86,87]. In line with this and to meet the objectives of our study, 15 open-ended primary questions with additional probing and follow-up questions were designed to increase the likelihood of rich responses, while ensuring the questions were not leading in order not to influence the answers provided by participants [88], and each question involved addressing only one topic at a time.

### 2.3. Study Design

The cross-sectional nature of our study focused on the period at the onset of the pandemic and during the lockdown periods in Bangalore. India went into lockdown on two occasions. In 2020, the country went into lockdown between the period March–May, and in 2021 between the period of April–June [89]. Due to low vaccine rates, the country was hit with the largest wave of the virus, resulting in the collapse of the hospital systems and, as a result, another lockdown period. Hence, our study primarily focuses on these two time periods.

### 2.4. Thematic Analysis Approach

#### 2.4.1. Tools and Ethics

Our study was approved by the University’s human studies ethics sub-committee. The video-call software employed had full end-to-end encryption and was compliant with privacy regulations including the GDPR/Data Protection Act 2018, with participants providing informed consent before any data collection. To minimize data storage for interviewees and since only the audio files were deemed necessary for transcription purposes and subsequent thematic analysis, only the audio files were stored on the lead researcher university’s secure IT services-provided cloud storage during the study. These were transcribed verbatim.

We employed thematic analysis as our method of qualitative analysis [90,91], which involves investigating, categorizing, and delineating ‘themes’ within the verbatim interview transcripts. A theme is commonly understood to represent a pattern in the qualitative response data [92] that encapsulates important details concerning the research objectives and signifies meaning [93]. In order to facilitate the codifying of the data for the thematic analysis, we employed established computer-assisted qualitative data analysis software [94]; specifically, ‘Delve’ software was used [95,96], as the software has powerful features to help streamline and simplify the qualitative analysis process and allows for subsequent filtering and comparison of codified themes.

#### 2.4.2. Thematic Analysis Phases

Our study employs the five distinct thematic analysis phases outlined by Braun and Clarke [93] in their landmark study. 

This includes data familiarization (Phase 1), and as pointed out in [93], the repeated reading of the textual data leads to better understanding and is a crucial first step to thematic analysis, as it allows the initial opportunity to identify patterns and meanings within the dataset. This phase also consisted of transcribing the interviews and organizing the transcripts in a readable format [97]. Phase 2 involves developing initial codes from the data, where a code is an elementary subset of the raw dataset and can be meaningfully interpreted [93]. A total of 90 codes were initially generated, and these encapsulate the interpreted essence of the raw data. Next, themes were created (Phase 3), with these codes being categorized and consolidated into themes, which signify recurring patterns within the dataset, as outlined in [98]. At this stage, themes relevant to the objectives of the study were identified as candidate themes. Subsequently, in Phase 4, all themes were evaluated for their relevance to ensure that they followed a coherent pattern and were further refined as necessary. During this step, some codes were moved to other themes as they were not consistent with the overall message, and the rationality of each theme concerning the whole dataset was considered [93]. Finally, in Phase 5, the themes were defined and a detailed analysis of each was performed. During the analysis, sub-themes (i.e., themes within a theme) and overarching themes were defined and mapped using Delve software. This helped provide structure to themes that were more complex in nature [93,98].

This final phase of the thematic analysis involves presenting the results of themes found within the data. This will be covered in the results and discussion sections. 

## 3. Results

**Figure 1:**
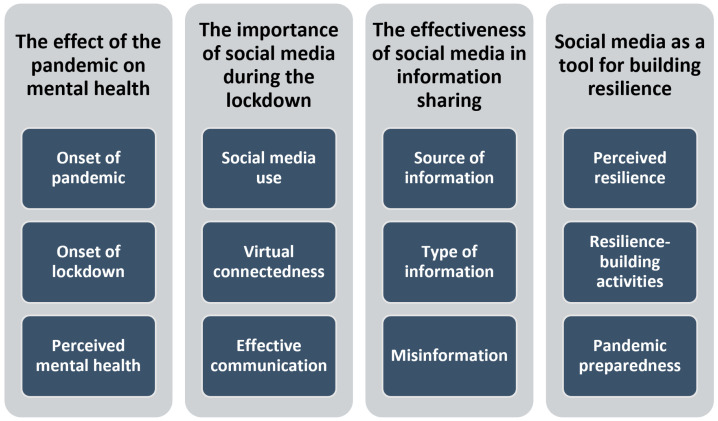
Primary themes and sub-themes.

The thematic analysis produced four primary themes: (1) the effect of the pandemic on mental health; (2) the importance of social media during the lockdown; (3) the effectiveness of social media in information sharing; and (4) social media as a tool for building resilience. Each primary theme is further classified into 3 sub-themes, as shown in Figure 1 below, with a total of 67 codes being identified for analysis. Some additional codes identified were not used as they were not relevant to the aims of this study.

In the following sub-sections, we present the key findings, with examples, across these themes. The names of all participants have been changed to protect their anonymity, and we make use of numeric codenames as they help conceal personal information [99].

### 3.1. Primary Theme 1: The Effect of the Pandemic on Mental Health

#### 3.1.1. Onset of Pandemic

This sub-theme covers the initial thoughts and reactions of participants to COVID-19. When asked to describe their initial thoughts on the pandemic, four out of the eight participants used words such as ‘apprehension’, ‘fear’, and ‘worrisome’ to describe their initial thoughts about COVID-19. Two participants did not expect the virus to reach India and two participants expected the virus to be temporary like other epidemic outbursts. As Participant 1 describes it, *“I did not anticipate COVID to be the worldwide phenomenon it turned out to be, I had categorized it as one of the epidemics that mushroom and then quickly concludes, like the Ebola or Zika virus. I did not fathom the impact of COVID on the world and society at large”*.

Six participants reported not anticipating the global effect of the pandemic.

#### 3.1.2. Onset of Lockdown

This sub-theme covers the sentiments related to the lockdown period. Six participants reported a positive reception to the lockdown. Three participants preferred the change of working from home during the lockdown; this is similar to the findings in a previous study by Wrycza and Maślankowski [100]. While two participants reported feelings of anxiousness and curbed freedom, in contrast, two other participants described the lockdown to be the best change in their lives.

For Participant 2, it was a chance to spend time with those he was closest to. He said, “To be honest, the early days of lockdown was a big lifestyle change. And I welcomed it with open arms, because I felt it broke the monotony of the way things were, and got to spend a lot more time with the people closest to me”.

While the initial reception of the lockdown was described positively, the next subtheme covers the prolonged effect of the lockdown on mental health.

#### 3.1.3. Perceived Mental Health

This sub-theme covers the perceived mental health of participants during the entire duration of the pandemic/lockdown. Four out of the six participants who reported a positive reception to the onset of the lockdown reported deteriorating mental health over time. Three participants described feelings of being ‘caged’, ‘curbed’, and ‘confined’. One participant in contrast reported no change in his mental health.

When asked if social media played a role in affecting their mental health, two participants reported that the use of social media influenced their mental health negatively. Participant 3 explains, “*Whatever time I did spend on Instagram, I would see people portraying a very beautiful version of their life, which is not necessarily the real version. And that would sometimes make me wonder, what am I doing wrong in my life?*”.

However, half the participants, in contrast, credited the use of social media as having a positive influence on their mental health. Two other participants reported no change in mental health after using social media.

### 3.2. Primary Theme 2: The Importance of Social Media during Lockdown

#### 3.2.1. Social Media Use

This sub-theme covers the general use of social media during the lockdown period of the pandemic. All participants reported an increase in smartphone screen time during the lockdown. All participants differentiated between various social media platforms and why they used them. 

As Participant 4 describes it, *“Social media is a very broad umbrella term. What TV was in the 1970s is today’s YouTube. Instagram is for checking up on your circle of friends, but also a source where you have micro dashes of entertainment. Twitter forces you to digest information in a very quick format and WhatsApp is much more of a communicative medium”*.

All participants used WhatsApp and it was the primary tool for communicating with friends and family. All participants used Instagram; it was described as a platform that provided entertainment as well as a means of staying in touch with an extended circle of friends. Six participants used YouTube and it was described as a source of news and entertainment. Four participants reported the use of Twitter to stay updated with the news. Two participants used Reddit to regularly explore personal interests.

#### 3.2.2. Virtual Connectedness

This sub-theme explored a range of topics related to the importance of staying connected during self-isolation. All participants reported using social media to stay connected with friends. Three participants indicated that they substituted in-person gatherings with friends for online virtual meetings and reported that without social media they would have felt lonely and isolated. Five participants reported an increase in feeling connected via social media. Participant 5 explains what life would be like without social media: “*I feel without social media, I could have felt more isolated. With social media, you’re still seeing what’s happening with your friends, so even though they’re not present physically, we were at least able to connect virtually*”.

For Participant 4, it was comforting to know that he was not doing it alone: “I was certainly on social media a lot simply because I could stay connected. I could at least feel part of the larger struggle. And I think that kind of suits your ailments in a way because you know that you’re not doing it alone”.

#### 3.2.3. Effective Communication

This sub-theme explored the means and importance of staying connected during isolation. Seven participants reported using video calls to communicate. Seven participants communicated using messaging services on social media. As Participant 2 put it, “*We’ve had video connectivity before but never really paid attention to it. It was only during the lockdown that we realized how much technology had evolved and how easy it is to communicate through social media*”.

For Participant 6, it was important for him to know that everyone was okay. “We used to have many video calls during the lockdown even my grandmother who had hearing issues used to join the call and I recall reassuring her that everything would be okay”.

### 3.3. Primary Theme 3: The Effectiveness of Social Media for Information Sharing

This theme captures the responses of participants related to the variety of health information encountered on social media.

#### 3.3.1. Source of Information

This sub-theme identifies the primary source of information during the lockdown. Participants were asked questions related to how they received information related to the pandemic as well as details relating to precautionary measures.

While two participants reported only using traditional media for information related to the pandemic, six participants, however, relied on social media, with two of those participants following trusted doctors while four others followed the social media accounts of trusted news outlets. Two participants credited the importance of following the right influencers as a key factor. Participant 1 pointed out the effectiveness of Twitter in narrowing down news about his locality. “*Through Twitter, I was able to get world news, central government news, state-related news and in some cases news about my quarter of the city, […] hyperlocal news*”.

#### 3.3.2. Type of Information

This sub-theme captures responses related to the type of health information content received by participants on social media. Seven participants reported receiving precautionary advice from social media. Information related to following protocols of social distancing and best practices such as the washing of hands and the importance of wearing a mask were all relayed via content on social media. In three instances, the participants reported a lack of trust in the information being dispersed by reputed organizations such as the CDC, UN, and WHO. As Participant 4 put it, “*In the beginning, the information that we were getting from the CDC and all the other major global health organisations was themselves not in line, this was not a very consistent approach to dealing with a pandemic*”.

#### 3.3.3. Misinformation

This sub-theme captures the perceived infodemic during the lockdown, and how it was identified. All participants reported encountering fake information on social media at some point. Two participants were not able to identify fake information online. Three participants reported disguised fake content to be the biggest factor in not being able to identify fake information. As Participant 2 points out, “*In social media fake news can be so well disguised that I feel that a lot of the time you’re just bound to believe it*”.

Five participants said that they were able to identify inaccurate information. They further added that independent research helped them identify the authenticity of the content they saw on social media. Half of the participants reported not taking everything at face value but instead viewing everything with a pinch of salt.

### 3.4. Primary Theme 4: Social Media as a Tool for Resilience

#### 3.4.1. Perceived Resilience

This sub-theme encapsulates participants’ conceptualization of resilience and their perception of social media’s influence on resilience. Each participant conveyed a perceived increase in resilience throughout the lockdown period. While two participants perceived an increase in resilience independent of external factors, all other participants attributed the influence of social media to improving resilience. 

As Participant 4 put it, *“Resiliency, for me, is being able to cope with my mental health and being grateful I was to be in this time where I have not only information of the welfare of my dear ones, but also, I could, for example, open up YouTube and follow steps and best practices from people all around the world, and listen to their coping mechanisms, which eventually was tailored into mine. Social media platforms, in that sense, have helped in building a lot of people up during that time”*.

Three participants credited watching others cope online, as well as knowing that they were not alone in their struggle, as a key factor in resilience building.

#### 3.4.2. Resilience-Building Activities

This sub-theme covers the distinct activities that helped interviewees in improving their resilience. All participants reported a change in their habits during the pandemic. Two participants explored creative avenues such as starting an Instagram account dedicated to art, as well as learning how to play the guitar online. Three participants reported being mindful of their diet based on suggestions made by influencers on Instagram, six participants found content related to home-based workouts/physical exercise as a means of coping with adversity. Participant 7, for instance, found the Instagram algorithms to be helpful, “*I believe when you have people close to you, sending you posts about mental well-being, the Instagram algorithm adapts and then provides suggestions of accounts that you could try and follow. So that gave me exposure to therapists and also to books that I could read about mental health*”.

Two participants found posts related to mental health, such as breathing exercises, and meditation to help build resilience. One participant reported going to therapy based on the content they were exposed to on Instagram.

#### 3.4.3. Pandemic Preparedness

This sub-theme highlights how social media was used as a tool for pandemic preparedness and response, and largely relates to social support facilitated via social media. Seven participants reported either helping or receiving help via social media. Six participants emphasized the crucial role of social media in disseminating vital information about resources, such as the availability of oxygen cylinders and hospital beds. As Participant 5 put it, “*When my mom was ill [with COVID-19], I got a lot of help* via *Twitter. I was able to secure oxygen cylinders, I was able to get medicines, and I was able to get beds all through social media. A lot of these pages were set up just to help. A lot of good people did a lot of good work*”.

Half the participants reported using social media to find hospital beds. Two participants used social media to help others financially. One participant used social media to donate plasma, while another used social media to help arrange food. Two participants said that social media helped save lives.

## 4. Discussion

Based on the in-depth interviews and qualitatively rich thematic analysis findings in our research sample (*n* = 8; thematic codes = 68), several key observations can be made with respect to experiences of social media-related resilience. 

*First*, social media was utilized as a tool for bolstering multiple types of community resilience and was also beneficial in building individual resilience. Our results suggest that social media was instrumental in influencing resilience-building activities such as pursuing hobbies, maintaining healthy diets, as well as physical exercise, particularly at a time when residents of Bangalore, a densely populated urban setting, were confined to their small accommodations. These results reinforce the findings reported in a study by Rosen et al. [101]. The pursuit of hobbies on social media, such as creating a dedicated art page, and learning how to play the guitar helped members maintain a positive outlook and fostered a sense of purpose [43,69] during a time of crisis. Maintaining a positive outlook has been previously shown to reduce the negative emotions caused by the pandemic [45] and having a sense of purpose has been shown to improve resilience [47]. Furthermore, similar to the work in [102], our findings also point to the influence of social media in encouraging community members to engage in positive health behaviors.

*Second*, our findings indicate that various interviewees engaged in sharing substantive resources during the lockdown. Resources such as monetary aid and providing food for those affected by COVID-19 were shared thanks to social media. The need for resources was broadcasted on social media platforms in the form of requests-for-help on Instagram stories and WhatsApp, thus enabling an environment where resources could be shared. Mano [17] argues that social media enhances feelings of social support as support can be received from various sources, such as organizations, social institutions, acquaintances, family, and friends, while other supporting studies suggest that by offering social support, community members can enhance their sense of belonging and integration with society, thereby nurturing a sense of community [103]. In a study of residents affected by a hurricane [41], a strong link between social support seeking and perceived positive resilience was suggested, and it can be postulated that the use of social media for social support could be viewed as a resilience-building factor during and in the aftermath of crises.

*Third*, in addition to providing a medium for social support, social media also fostered an environment for prosocial behavior. For instance, the number of available hospital beds in India had fast depleted during the pandemic, and this was coupled with the fact that oxygen cylinder production failed to keep pace with the increased demands [67,104]. During the collapse of the hospital systems, however, an abundance of help came from crowdsourcing via social media channels such as Twitter, Facebook, WhatsApp, and Instagram [66]. At the height of the pandemic, social media was used as a temporary database of sorts, and the findings of this study lend clear support to the notion that social media provided crucial life-saving information. This included information on available hospital beds and information on oxygen cylinder manufacturers. In addition to this, community members offered ‘plasma donations’ after recovering from COVID-19. Prosocial acts such as this have been shown before to improve well-being and life satisfaction [70,71]. Prosocial behavior arises from having a shared identity with someone going through a difficult time [69] and it can be argued that during the lockdown, given that all members of the community were experiencing the same hardships, social media provided an environment for community members to engage in prosocial behavior such as ‘financial donations’, ‘plasma donations’, and it also provided a platform for the sharing of information on crucial resources. Thus, the timely exchange of information on social media was an important aspect of building resilience.

*Fourth*, the results of this study suggest that social media was important in reducing feelings of loneliness as the community members were able to foster a sense of belonging and connectedness by using social media and messaging services. This is in support of studies in the past that have shown active engagement on social media is a coping mechanism for those who are isolated [105,106], allowing them to communicate and stay connected, thus building the perception of community resilience. This is further supported by Tkáčová et al. [107], who examined high school students and showed that increased connectivity on social media improved the overall mental well-being of the participants studied. Social media helped overcome the physical barriers of isolation [17] and helped fulfill the fundamental need for belonging during isolation [30]. Our results also suggest that watching others cope online helped members feel less isolated and feel they were part of the larger struggle. This is congruent with the findings of Rosen et al. [101] and Moore and March [108], who argue that factors such as media connectedness and offering social support via social media can, in some cases [109] during a time of crisis, help minimize feelings of loneliness.

*Fifth*, during the codifying phase of the thematic analysis, we observed that interviewees used rich expressions to refer to specific themes, such as ‘apprehension’ and ‘fear’, including strongly experienced feelings of being ‘caged’, ‘curbed’, and ‘confined’ during various recollections of pandemic experiences. While we have not explored this further, as it is outside of the immediate scope of our study, there is a body of prior evidence that suggests specific language word use and related emotional word sentiments strongly correlate with unique affective traits, themes, events, and experiences (e.g., [110]). Related big data word analytics tools have increasingly been developed to measure higher-level concepts, in text-based social media data at scale, such as stress [111], affective states [112], and sentiment and emotional expressions [113,114], which have numerous potential applications including as digital epidemiology tools, particularly in densely populated urban areas [36], allowing hotspots of negative emotions and stress or resilience to be tracked. Nevertheless, many text analysis tools are based on machine learning top-down approaches [113,115], while we believe there is considerable potential for leveraging small-sample, in-depth qualitative methods to inform, integrate, and co-create digital epidemiology tools through bottom-up approaches based on experiences and authentic language use of individuals on the ground, as opposed to relying on top-down approaches (see [116]). The advantage of bottom-up approaches is an emphasis on semantics and an in-depth qualitative understanding of social media user-generated content and the users directly engaged in producing content, rather than narrow and purely machine learning big data approaches, as we outline further in the future research sub-section.

Overall, we found that social media played a substantive role in modulating resilience-related experiences among participants who were based in a hard-hit COVID-19 urban community. Various studies in the past have analyzed factors contributing to building resilience during and in the aftermath of a disaster (see [34,117,118,119,120]), and of the several factors identified, ‘communication’, ‘connection’, and ‘information sharing’ are repeatedly recognized resilience-building factors. The results of our study indicate that social media played a pivotal role in not just addressing the aforementioned factors, but also in its application as a medium for disaster response, i.e., its role in improving social capital as well as encouraging prosocial behavior in the form of community support in an urban setting during a time of crisis.

### 4.1. Limitations

There are several limitations in this study. To begin with, our study was conducted over one year after the onset of the pandemic lockdown period, which adds to the possibility of various biases, especially recall bias as outlined in [121,122]. Second, the data collected only represents a small size of the members of the specific urban community of Bangalore; however, our intent was strictly not to generalize [80] any findings but to uncover deeper richer qualitative insights, which is in line with similar types of interview research (e.g., [123]). To add to this, the findings in this study may not be consistent with other regions which may have varying environments. Third, this study is most likely subject to gender bias as unfortunately seven of the eight participants who took part in this study were male. Fourth, the participants studied were highly educated and well versed in technology; therefore, the findings are not meaningful with respect to drawing conclusions for less digitally literate individuals, while for instance, members of the adolescent or older generation were not included. Prosocial tendencies may also vary across different samples. Relatedly, our sample was biased towards ‘white-collar’ professionals. Nevertheless, as per the 2011 Indian census, these types of jobs represent a significant proportion of professions in the Bangalore urban district [74].

### 4.2. Future Research

To the best of our knowledge, our study is the first to probe into the impact of social media on building pandemic resilience within Bangalore’s urban community, while more broadly we contribute towards filling the paucity of research on utilization of social media as an instrument for fostering resilience. Given the insights gleaned from this investigation, there is a fertile ground for potential future research. Further exploration should consider expanding the variety and sample sizes across the demographic range to include different ages, genders, professions, and social classes, thereby painting a more comprehensive picture of social media’s impact on resilience. Moreover, comparative studies could be undertaken between different urban regions and rural communities to assess differences and similarities in digital resilience-building strategies, including across states and countries. Longitudinal investigations could yield valuable insights into the lasting impact of social media-facilitated resilience in post-pandemic urban communities. The intersection of misinformation and resilience building through social media also remains a largely uncharted area, while research that interrogates the role of different social media platforms in facilitating resilience could be beneficial in shaping platform-specific pandemic response strategies. 

Finally, even though with eight participants the sample of interviewees in this study was rather small, with regards to the qualitative depth and richness of the data and over sixty codified expressions relating to the primary themes and sub-themes in the thematic analysis, these qualitative observations provided an important baseline context of word use and expressions across pandemic experiences. In line with calls for more research focusing on highly qualitative approaches that can inform computational and digital methods [39,124], we see the potential for increasingly integrating in-depth qualitative approaches (i.e., small data) with big data in a bottom-up manner (see [116]) for the development of text analytics social media digital epidemiology tools (e.g., [36]). This could be achieved through using qualitative analysis results from thematic analyses or concept mapping, down to specific word and expression use, to feed into the construction of dictionaries or richer semantic models such as formal ontologies [111] and could even involve participants in tool co-design [125] with inclusivity and qualitative outputs being situated within relevant respective communities [126], but is outside the scope of our current study. 

## 5. Conclusions

In this study we elucidate the potential of social media as a crucial tool for fostering resilience during times of crises, specifically the COVID-19 pandemic within Bangalore’s urban landscape. Our findings indicate that social media played an instrumental role in facilitating effective communication, nurturing community ties, and disseminating essential information within an urban space. Furthermore, social media spurred an environment conducive to prosocial behavior, a recognized resilience amplifier, especially during the collapse of the hospital systems in Bangalore, where social media played a key role in sharing lifesaving information about the availability of hospital beds and oxygen cylinders, including plasma and financial donations. Our research, the first of its kind in the Bangalore context, underscores the multifaceted influence of social media in a pandemic setting. It contributes to the larger discourse on digital epidemiology and urban health by shedding light on spatial–digital interactions during the lockdown. However, this exploration is but an initial step in comprehending the complexities of social media use in times of crisis. Further studies are needed to build upon these findings, to evaluate the long-term implications of these digital interactions, and to create strategies that can harness social media’s potential for building resilience in diverse urban health contexts. There is also much potential for embedding small-sample and in-depth qualitative methods in informing, integrating, and co-creating digital epidemiology tools bottom-up, rather than top-down. We intend for our work to further emphasize the importance of a balanced perspective on social media during crises, moving beyond its drawbacks to recognize its capacity for building resilience within communities. We also hope to call on policymakers, urban planners, researchers, and social media platforms to acknowledge this potential and integrate it into their pandemic response strategies and digital epidemiology tools where appropriate.

## Data Availability

The data presented in this study are available upon request from the corresponding author. The data are not publicly available due to privacy and ethical restrictions.
